# Causes for Medical Errors in Obstetrics and Gynaecology

**DOI:** 10.3390/healthcare11111636

**Published:** 2023-06-02

**Authors:** Désirée Klemann, Maud Rijkx, Helen Mertens, Frits van Merode, Dorthe Klein

**Affiliations:** 1 Department of Gynaecology and Obstetrics, Maastricht University Medical Centre+, 6229 HX Maastricht, The Netherlands; 2 Care and Public Health Research Institute, Maastricht University, 6200 MD Maastricht, The Netherlands; 3 Department of Plastic, Reconstructive and Hand Surgery, Maastricht University Medical Centre+, Maastricht University, 6229 HX Maastricht, The Netherlands; 4 Executive Board, Maastricht University Medical Centre+, Maastricht University, 6229 HX Maastricht, The Netherlands; 5 Maastricht University Medical Centre+, Maastricht University, 6229 HX Maastricht, The Netherlands; 6 Department of Clinical Epidemiology and Medical Technology Assessment, Maastricht University Medical Centre+, 6228 HX Maastricht, The Netherlands

**Keywords:** medical errors, adverse events, gynaecology and obstetrics, healthcare quality, safety, systematic review, medical record review

## Abstract

**Background:** Quality strategies, interventions, and frameworks have been developed to facilitate a better understanding of healthcare systems. Reporting adverse events is one of these strategies. Gynaecology and obstetrics are one of the specialties with many adverse events. To understand the main causes of medical errors in gynaecology and obstetrics and how they could be prevented, we conducted this systematic review. **Methods:** This systematic review was performed in compliance with the Prisma 2020 guidelines. We searched several databases for relevant studies (Jan 2010–May 2023). Studies were included if they indicated the presence of any potential risk factor at the hospital level for medical errors or adverse events in gynaecology or obstetrics. **Results:** We included 26 articles in the quantitative analysis of this review. Most of these (*n* = 12) are cross-sectional studies; eight are case–control studies, and six are cohort studies. One of the most frequently reported contributing factors is delay in healthcare. In addition, the availability of products and trained staff, team training, and communication are often reported to contribute to near-misses/maternal deaths. **Conclusions:** All risk factors that were found in our review imply several categories of contributing factors regarding: (1) delay of care, (2) coordination and management of care, and (3) scarcity of supply, personnel, and knowledge.

## 1. Introduction

Much research is performed on ‘quality of healthcare’. Most recent definitions of ‘quality of healthcare’ are provided by the European Commission and the World Health Organization (WHO) [1,2,3]. Both the European Commission and the WHO describe ‘quality of care’ as the degree to which health services for individuals and populations increase the likelihood of desired health outcomes, working from evidence-based professional knowledge. Quality services should, according to the European Commission and WHO, be effective, safe, and people-centered and, therefore, must be timely, equitable, integrated, and efficient.

A broad range of quality strategies, interventions and frameworks have been developed with the aim of facilitating a better understanding of health systems and improving the quality of healthcare. The strategies can be summarized into (1) system-level strategies, (2) institutional/organizational strategies, and (3) patient-level strategies [4]. One broadly implemented strategy at the organizational level is reporting adverse events [5]. Often, adverse events function as a starting point for root cause analysis to identify direct and indirect causes of safety incidents [6]. These definitions and strategies presume a relation between ‘quality’, ‘safety’, and ‘risk’ of occurring ‘medical errors’.

An adverse event is defined as an unwanted outcome of (delayed/lacked) medical treatment. Adverse events and medical errors imply that a person, situation, system, or a combination of these factors has caused health damage [7]. The “Harvard medical practice” study found that 3.7% of all hospital admissions led to adverse events, while the “quality in Australian healthcare study” identified adverse events in 16.6% of admissions. Half of these adverse events were preventable [8]. Recent data from Liu et al. showed that the highest numbers of adverse events are seen in the medical specialties: general surgery, orthopaedics, and obstetrics/gynaecology [9].

To understand the main causes of medical errors in gynaecology and obstetrics, how they can be prevented, and how the quality of care can be improved, we conducted a systematic review. The goal of this review was to identify the direct and indirect causes of medical errors and mistakes related to one of the top three medical specialties, obstetrics and gynaecology, due to the high number of adverse events and the potential high impact of adverse events in this specialty. Understanding the direct and indirect causes of medical errors can lead to improvements in the quality of care.

## 2. Methods

### 2.1. Literature Search

This systematic literature search was electronically performed for relevant studies in PubMed, EMBASE, web of knowledge and the Cochrane Library. For this search strategy, the following MeSH- and non-MeSH terms were used: medical error, medical mistake, adverse event, risk management, health care quality assessment, health care quality, access, evaluation, gynaecology and/or obstetric. The following limits were added to these terms: full text, 2010–2023, language restriction in Dutch and English, and the presence of gynaecology and/or obstetrics in the title or the abstract. Since maternal care was part of the millennium development goals report in 2010 and the increased attention on maternal care ever since, we limited our search to the period from 2010 until 2023. The researchers MR and DoK have performed all searches. The final search was performed on 11 May 2023. For the specific search terms, including used limits and the number of articles for each database, see Appendix A. The systematic review was performed respecting the PRISMA 2020 guidelines [10].

### 2.2. Eligibility Criteria

All included articles indicate the presence of any potential risk factor at the hospital level for medical errors or adverse events in gynaecology or obstetrics. Only full articles were included. Letters, abstracts and review studies were excluded. All articles that did not contain information about the cause of medical errors or adverse events or articles exclusively describing patient-related risk factors were excluded from this literature review.

### 2.3. Study Selection

After completion of the search, all articles were independently screened on title by two researchers (DeK, MR). Disagreements at this stage were dealt with by discussion and consensus. If disagreement was maintained, a third independent researcher (DoK) was involved in the final decision. After the completion of screening on the title, the remaining articles were further analyzed on the abstract and full text by the same three authors (DeK, DoK, MR).

### 2.4. Data Extraction

The included articles were analyzed on the title, author, year of publication, study setting, study design, study population, study size, results, factors, and conclusion. After completion of the extracted data, this information was clustered in a summary of findings table (Supplementary table). Data extraction was conducted by all three authors separately (DeK, DoK, MR). All articles were analyzed using the Prisma checklist [10]. Again, disagreements were dealt with by discussion and consensus. In order to provide an overview of the factors contributing to adverse events, near-misses and medical mistakes, results were summarized per category according to the framework provided by Tello et al. (2020) [11].

## 3. Results

### 3.1. Results of the Search and Study Selection

**Figure 1:**
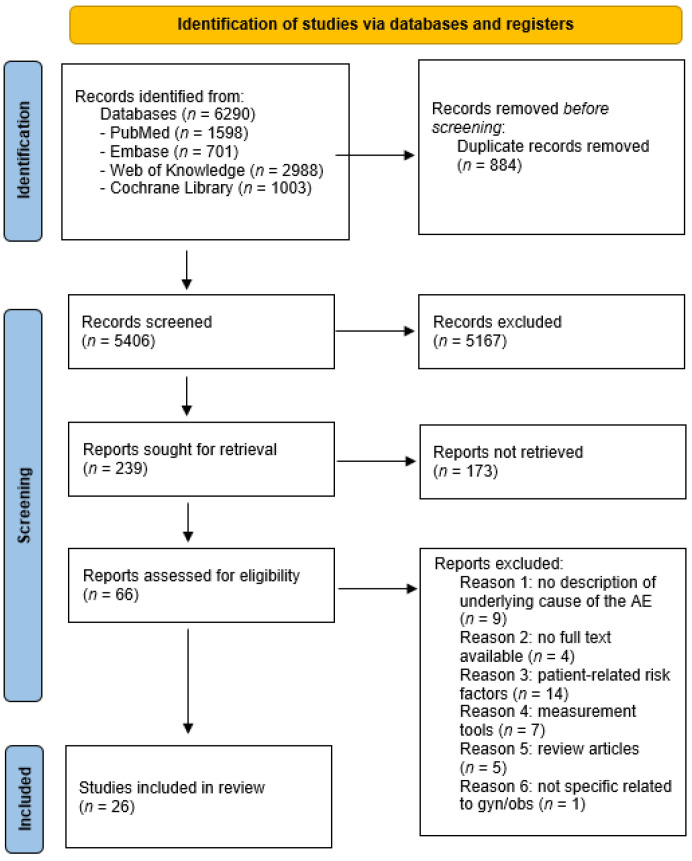
Flowchart of the literature search and selection of studies.

After completing the search, 6290 articles were identified before deduplication. Following manual and automatic deduplication (N = 884), 5406 articles remained for further analysis. After screening the title, 239 records remained for further analysis on the abstract. After reviewing the abstracts, an additional 173 articles were excluded as they did not meet the inclusion criteria. A total of 66 full-text articles were independently analyzed by three researchers (DeK, DoK, MR) for eligibility, resulting in 26 articles for quantitative analysis in this review. A flowchart and an overview of all exclusion details can be found in Figure 1.

### 3.2. Quality Criteria

The quality of the included studies was evaluated using the criteria developed by Worster et al. for assessing the quality of MRR studies [12]. Quality assessment was performed in duplicate by DeK and DoK. The assessment used rating categories of “present” or “missing”, which were transformed into 1’s and 0’s and added together for a score between 1 and 15. Studies with scores between 0 and 5 were considered weak; those with scores between 6 and 10 were deemed reasonable, and studies with scores between 11 and 15 were classified as good. The results are shown in Table 1.

### 3.3. Included Studies

Patient populations or clinical conditions that were studied included pregnant and postpartum women (within 42 days of termination of pregnancy) admitted to the obstetric department and also maternal near-miss and maternal death cases. The studies that were included varied in study center extent and study size (1 hospital versus nationwide inclusion; sample sizes varied between 18 and 27,916). The studies were performed in 18 different countries (see Supplementary table).

Most of the studies were cross-sectional studies (*n* = 12), case–control studies (*n* = 8) or cohorts (*n* = 6). 21/26 studies were based on medical record reviews. All studies were quantitative studies except two, which combined qualitative and quantitative research by means of interviews with healthcare professionals and patients. Table 1 shows the quality assessment of the included articles, which were considered weak (*N* = 9) and reasonable (*N* = 12) quality. For five articles, the criteria were not applicable.

### 3.4. Definitions

Only seventeen of the twenty-six articles explicitly stated a definition of the patient population or clinical conditions that were included and/or the outcome that was measured (for example, ‘adverse event’ or ‘maternal near-miss’). If provided, definitions varied between these articles. In 12 articles, the WHO definitions and criteria were followed. The WHO defines an *adverse event* as an injury related to medical management, in contrast to complications of a disease. Most studies include cases of *maternal near-misses*, which is defined by the WHO as ‘a woman who nearly died but survived a complication that occurred during pregnancy, childbirth or within 42 days of termination of pregnancy’ [39]. Both adverse events and maternal near-misses may be preventable or non-preventable. Table 2 provides an overview of definitions used in the included articles.

### 3.5. Findings

Most of the included studies (*n* = 21) were medical record reviews. All studies except two were quantitative studies. These combined qualitative and quantitative research by means of interviews with healthcare professionals and patients. All studies were related to obstetric care and *maternal near-misses*. Supplementary table provides a summary of the findings table of all included studies.

In 25/26 studies, cases of maternal or neonatal near-misses, maternal deaths, or adverse events were selected using different definitions and selection criteria. After selection, a retrospective (medical record) review was performed by an internal and/or external committee. Most studies provide an overview of factors that contributed to the near-misses, maternal deaths or adverse events. One of the most commonly reported factors is *delay in healthcare*. In addition, *availability of products* (such as medication and blood products), *availability of trained staff*, *team training* and *communication* are often reported to contribute to near-misses/maternal deaths. In Table 3, we provide a summary of the most commonly reported contributing factors, categorized per quality-of-care mechanism, according to the framework provided by Tello et al. [11]. The following mechanisms are described: *patient-related factors*, *clinical practice*, *emergency medicine*, *management*, *workforce*, *pharmaceuticals*, *medical products*, *health facilities,* and *information systems*. The percentage indicates the relative number of the studied cases in which the contributing factor played a part. For example, in the study of Aikpitanyi, delay in commencing treatment played a part in 27.8% of all cases analyzed.

Table 4 summarises and categorises the contributing factors according to the level of healthcare in which they occurred, including individual healthcare workers (nurses or doctors), teamwork, or the healthcare system in which the team and the individuals cooperate. Some factors may occur on multiple levels (such as *delay*). Not all factors described in Table 3, are categorised in Table 4, because of insufficient information in the primary studies to determine the level of healthcare in which these factors occurred (for example, *monitoring problems*, *inadequate preparation* and *medication errors*).

## 4. Discussion

The aim of our review was to identify the direct and indirect causes of medical errors and adverse events in obstetrics and gynecologic practice. Our review included 26 studies from the last 13 years concerning the direct and/or indirect cause(s) of medical errors in obstetrics. The included studies are cross-sectional studies (*N* = 12), case–control studies (*N* = 8), and cohorts (*N* = 6), mainly based on retrospective medical record reviews. Maternal deaths and maternal near-misses were frequently used to select cases for medical record reviews.

The included studies were performed in 18 different countries and under different conditions, including developed and developing countries.

Table 3 summarizes the “quality of care” mechanisms that were frequently found to be a contributing cause to the onset of medical errors and adverse events. All of the risk factors identified in this review imply several categories of direct and indirect factors regarding: (1) *delay of care*, (2) *coordination and management of care* and (3) *scarcity of supply, personnel, and knowledge*. These factors occurred at both the level of individual healthcare workers (such as *not following protocol*, *delay in decision making*) and at a system level (*no protocol available*, *lack of staff and equipment*).

Although most included studies describe the same types of risk factors for errors and adverse events, it is important to interpret these results with knowledge of the (local) circumstances of the studies, such as socio-economical, geographical, cultural and financial factors.

Studies conducted in developing countries often found a *lack of supply*, *lack of blood products*, *non-functional IC-units* and *non-availability of medication* as causes for errors and adverse events [14,15,27,28,35,36,37]. Although these factors do not seem to apply to developed countries, recent circumstances, such as the COVID-19 pandemic and war in Europe and Asia, have shown the vulnerability of the current health and medical systems. The import of medication and supply from abroad is currently under pressure.

The same applies to the factor *a lack of (qualified) personnel*, which is mainly described in low-income countries [17,29,31,35]. However, with an imminent shortage of healthcare personnel worldwide, developed countries will be threatened by this factor as well.

Furthermore, studies in both high- and low-income countries found *second and third delay* to be risk factors for medical errors and adverse events. *Second delay* is related to reaching an appropriate health facility, and *third delay* occurs once the patient reaches the health facility and is waiting to see a medical professional. The proportion of cases in which *treatment delay* had a part ranged from 0.9% [22] to 42.9% [29]. In developing countries, *second and third delay* occurs due to a lack of supply, ambulances and poor infrastructure [14,15,19,22,27,29,30,32,35]. In developed countries, *second and third delay* appeared to occur as a result of delayed referral between first-, second- and third-line healthcare systems, for example, in countries where home birth is still common [23,24]. In addition, a *lack of teamwork and communication* at the moment of referral increases the risk for errors and adverse events [16,18,20], even as a *lack of continuity and coordination* of antenatal and obstetrical care [31]. Lastly, *shortage of information* or an *incomplete medical file* also increases the risk of medical errors and adverse events [18]. This underlies the importance of an integral, cross-institutional medical file, which is currently not available in most developed countries due to privacy laws.

An additional important insight from the verification is that there is a lack of unambiguous terminology and/or definitions in the field of near-misses, complications, (medical) errors or root cause analysis. Table 2 shows that only 17 (of 27) studies provide a definition of these terms. In 9 studies, the definitions refer to criteria from the World Health Organization (WHO). To gain homogeneity, we strongly recommend that future research conform to these definitions using the WHO criteria.

This systematic review has some limitations. Disadvantages of medical record reviews are the risk of selection bias, hindsight bias and the fact that the reliability of the results depends on the quality (completeness/readability) of the included medical files and the experience of the abstractors. It is not clear against which standards/guidelines the files have been tested and whether there are differences in standards between high- and low-income countries.

Furthermore, the described factors remain quite superficial qualifications of context and situations and often did not expose root causes of adverse events and medical errors.

Fur multiple factors, such as *delay of care* and *communication*, a retrospective analysis comes with a high risk of hindsight bias and is judged in the eye of the beholder. While it may seem logical in retrospect that a quicker response or referral could have led to better outcomes, it is essential to question whether healthcare workers may have misinterpreted the available knowledge of the patient at the time; therefore, *incorrectly* referred or treated too late.

In addition, when a medical file is retrospectively judged, certain cause-and-effect relations are supposed and attributed to a healthcare worker, while it might have been the healthcare system that allowed the adverse event to occur. For example, if a patient in labor is referred ‘too late’ to a hospital, this might be due to a personal mistake by the healthcare worker. However, if the healthcare system was set up differently, the chance that this error would occur would be different. For example, in a healthcare system where all deliveries have to take place in hospitals, the chance that this error would have occurred is probably much smaller.

As mentioned in the introduction, quality improvement strategies have been developed on different levels: (1) system-level strategies, (2) institutional/organizational strategies and (3) patient-level strategies [4]. Unfortunately, medical record reviews focus on the health situation of the patient and do not provide any information regarding the local health system and possible factors of influence, such as the circumstances on the work floor at the moment of the incident, for example, workload, supply of material, working atmosphere, current lack of staff, etc. Medical record reviews might be deficient in identifying environmental circumstances that allow errors to occur, possibly leaving large amounts of information underexposed. This information could be obtained by, for example, interviewing staff and direct observations of patient care.

Although the registration of adverse events contributes as a signaling system for quality of care, a more in-depth analysis is essential to take preventive measures on a system or institutional level.

In most developed countries, ‘clinical audits’ are used to describe a process of assessing clinical practice against standards. Interviews of involved healthcare providers and patients are part of this and may improve the knowledge regarding risk factors at a system level. Analytical methods such as the Functional Resonance Analysis Method (FRAM) [40] are developed to provide insight into how healthcare professionals work together under complex circumstances and the ways in which they must adapt to fluctuations. Using a method like FRAM might improve the insight into causes for errors in a broader context, including the reality of the workplace.

## 5. Conclusions

This systematic review regarding the direct and indirect causes of medical errors in gynaecology and obstetrics has led us to 26 studies performed in 18 countries, mostly based on reported adverse events and medical record reviews. The findings provide insight into general and circumstance-specific, direct or indirect causes for adverse events and errors, such as (1) *delay of care*, (2) *coordination and management of care* and (3) *scarcity of supply, personnel, and knowledge*. With an eye to the future and an imminent shortage of healthcare personnel worldwide, these findings should be taken seriously by healthcare developers and should encourage changes at the system level in order to keep healthcare available, safe and of a high standard for every patient. 

In order to make preventive measures to avoid future errors, we would advise further research concerning the onset of medical errors or near-misses in relation to the healthcare system, workplace behaviour and safety/quality of healthcare. Therefore, we should analyse the healthcare system in which frequent errors occur in a broader context, including the reality of the workplace. The analytical method called Functional Resonance Analysis Method (FRAM) is an example of a method that provides insight into how healthcare professionals work together under complex circumstances and the ways in which they must adapt to fluctuations. With this method, important steps, supplies, personal staff and mutual interactions between these actors can be mapped, and preventive measures might be taken to avoid future adverse events and medical errors.

## Figures and Tables

**Table 1:** Quality assessment of included studies [12]. 1 = Present, 0 = missing.

	Abstractors Training	Selection Criteria	Variable Definition	Abstraction Forms	Performance Monitored	Blind to Hypothesis	IRR * Mentioned	IRR * Tested	MR Identified	Sampling Method	Missing Data Management	Institutional Review Board	Total Score
Aibar [13]	1	1	1	1	0	1	0	0	1	1	1	1	9
Aikpitanyi [14]	0	1	0	1	0	0	0	0	1	1	0	1	5
Benimana [15]	0	1	1	1	0	0	0	0	1	1	0	1	6
Carvalho [16]	0	1	1	1	0	1	1	0	0	0	0	1	6
David [17]	0	1	1	1	0	0	0	0	1	1	0	1	6
Florea [18]	No MRR, criteria inapplicable												
Habte [19]	1	1	1	1	1	0	1	1	0	1	0	1	9
Hadad [20]	1	1	1	1	0	0	0	0	0	0	0	1	5
Huner [21]	No MRR, criteria inapplicable												
Iwuh [22]	0	1	1	1	-	-	-	-	1	1	0	0	5
Jensen [23]	No MRR, criteria inapplicable												
Johansen [24]	0	1	1	0	0	0	0	0	1	1	0	1	5
Johansen [25]	0	1	1	1	0	0	0	1	1	1	1	1	8
Kalisa [26]	1	1	1	0	0	0	0	0	1	1	0	0	5
Kasahun [27]	1	1	1	1	0	0	0	0	1	1	0	1	7
Kulkarni [28]	1	1	1	1	1	0	0	0	1	1	0	1	8
Mahmood [29]	0	1	0	1	0	0	0	0	1	1	0	0	4
Mawarti [30]	0	1	1	0	0	0	0	0	1	1	0	0	4
Mulongo [31]	No MRR, criteria inapplicable												
Nassoro [32]	0	1	0	0	0	0	0	0	0	1	0	1	3
Neogi [33]	0	1	1	1	0	0	0	0	1	1	0	1	6
Saucedo [34]	0	1	1	1	0	0	0	0	1	1	1	1	7
Sayinzoga [35]	0	1	0	0	0	0	0	0	1	1	0	0	3
Sorensen [36]	0	1	1	1	1	0	1	0	1	1	0	1	8
Wasim [37]	No MRR, criteria inapplicable												
Zewde [38]	1	1	1	1	0	0	1	0	1	1	0	1	8

* Inter observer reliability.

**Table 2:** Terms and definitions used in each article.

Author	Terms Used	Definition Used
Aibar [13]	Patient safety incident	Any event or circumstance that caused or could have caused unnecessary harm to a patient.
	No harm incident	Any unforeseen and unexpected event recorded in the medical record that did not cause harm to the patient but which, under different circumstances, could have been an accident or an event that, if not discovered or corrected in time, could imply problems for the patient.
	Adverse event	Any unforeseen and unexpected accident recorded in the medical record that caused injury and/or disability and/or prolonged the hospital stay and/or led to death, which was the result of health care and not the patient’s underlying condition.
Aikpitanyi [14]	Maternal death	No definition provided
Benimana [15]	Maternal near-miss	Refers to WHO criteria
	Maternal deaths	Refers to WHO criteria
Carvalho [16]	Three delays	Refers to WHO criteria
David [17]	Near-miss cases	Refers to clinical criteria for identification of near-miss (e.g., eclampsia, severe hemorrhage, severe sepsis, uterine rupture and severe malaria).
Florea [18]	Averse events	No definition provided
	Incidents	No definition provided
	Near-misses	No definition provided
Habte [19]	Maternal near-miss	Refers to WHO criteria
Haddad [20]	Severe maternal morbidity	Refers to WHO criteria
Hüner [21]	Adverse events	A catalogue of criteria or events was developed based on international research findings from scientific studies in two project meetings and interprofessional focus groups.
Iwuh [22]	Maternal near-miss	Refers to WHO criteria
Jensen [23]	Adverse health outcomes	No definition provided
	Clinical performance	TeamOBS-PPH score
Johansen [24]	Adverse events	No definition provided
	Serious outcomes	No definition provided
	Serious adverse events	An injury was regarded as serious when it had serious consequences on the patient’s disease or disorder; or if it caused serious pain or reduced self-realization in the short or long term
Johansen [25]	Serious adverse events	Three categories were described (birth asphyxia, shoulder dystocia and severe PPH)
	Adequate obstetric care	Healthcare is in accordance with clinical practice based on Norwegian national and local obstetric guidelines.
Kalisa [26]	Maternal near-miss	Refers to WHO criteria: a woman who almost died but survived a complication during pregnancy, childbirth or within 42 days after the termination of pregnancy.
	Severe maternal outcome	Maternal near-miss and maternal deaths combined
Kasahun [27]	Maternal near-miss/severe maternal morbidity	Refers to WHO criteria and states that the terms maternal near-miss and severe maternal morbidity are used interchangeably.Operational definition: maternal near-misses (severe maternal morbidity) is women who are admitted with either of the following obstetric diagnoses: severe preeclampsia, eclampsia, severe hemorrhage, dystocia (defined in the current study as uterine rupture, impending uterine rupture like prolonged labor with previous cesarean section, and emergency C/S delivery), severe anemia (<6), sepsis (puerperal sepsis, chorioamnionitis and septic abortion).
Kulkarni [28]	Near-miss obstetric event	Refers to WHO criteria. Near-miss obstetric event concerns a woman who nearly died as a result of a complication that occurred during pregnancy, childbirth or within 42 days of termination of pregnancy. Clinical criteria near-miss events were defined as any near-miss event related to a specific disease entity, while management-based near-miss events and organ-system dysfunction-based near-miss events were defined according to the near-miss approach outlined by WHO.
Mahmood [29]	Maternal deaths	No definition described
Mawarti [30]	Maternal deaths	Refers to WHO criteria
	Maternal near-miss	Refers to WHO criteria
Mulongo [31]	Maternal near-miss	Refers to WHO criteria
Nassoro [32]	Maternal deaths that occurred due to haemorrhage	No definition described
Neogi [33]	Stillbirths	Any baby born dead after the 24th week of pregnancy.
Saucedo [34]	Pregnancy-associated deaths	All deaths of women while pregnant or within one year of the termination of pregnancy, regardless of the cause of death.
Sayinzoga [35]	Maternal deaths	No definition described
Sorensen [36]	Maternal death	No definition described
Wasim [37]	Maternal deaths	Refers to WHO criteria
	Maternal near-miss	Refers to WHO criteria
Zewde [38]	Severe maternal outcome	Combination of maternal deaths and maternal near-miss
	Maternal near-miss	Refers to WHO criteria

**Table 3:** Summary of contributing factors to adverse events or medical (near)misses and maternal deaths. This table describes, per quality-of-care mechanism, the contributing factors described per study. Percentages reflect the relative number of cases (from this study) in which this factor contributed to an adverse event.

Quality of Care Mechanism	Study	Results	Percentages
Patients	Iwuh [22]	Patient education (lack of information)	6.25
Clinical practice	Aikpitanyi [14]	Delay in commencing treatment	27.8
	Benimana [15]	Diagnostic delays	41.3
		Therapeutic delays	5.8
	Florea [18]	Protocol	5.9
		Nursing resources	0.2
		Physician resources	1.7
		Other personnel	0.7
		Equipment/resources	6.9
		Records/results	14.5
		Staff communication	10.0
		Patient/family communication	1.6
		Delay	1.0
	Haddad [20]	Lack of trained staff	5.1
		Difficulty in monitoring	8.1
		Delay in diagnosis	5.6
		Delay in starting treatment	6.5
		Delay in referral/transfer of the case	5.2
		Improper management of the case	21.8
	Iwuh [22]	Not managed at the level of care that was needed	20.5
		Clinical assessment (diagnosis), Problem recognition	4.5
		Delay in referring	0.9
		Managed at inappropriate level	0.9
		Monitoring problems	13.4
	Johansen [24]	Failure in surveillance	36
		Failure in diagnostics	17
		Failure in operative delivery	8
		Failure in resuscitation	2
	Sayinzoga [35]	Lack of skilled staff	
		Insufficient diagnostic means	
		Inadequate monitoring of labour (use of partograph)	
		Delay in recognising the complication or administering the correct treatment	
		Insufficient follow-up in post-operative or postpartum period	
		No respect for asepsis	
		Not following protocol	
		Inadequate resuscitation	
		Insufficient follow-up of anaesthesia induction	
		Insufficient pre-operative preparation	
		Poor quality of antenatal care visit	
	Sorensen [36]	Training of staff insufficient	
	Habte [19]	Poor birth preparedness and poor complication readiness	85.2
	Johansen [25]	Delay in decision to operate	8
		Delay in decision to delivery time	20
		Failure monitoring/Misinterpretation CTG	13
		Medication error	56.2
	Nasorro [32]	Delay in managing uterine atony	17
	Carvalho [16]	Inadequate prenatal care: improper conduct with patient	5 neonatal near-miss/1 death
	Huner [21]	Peripartum therapeutic delay	44.32
		Diagnostic error	36.36
		Inadequate birth position	34.09
		Medication error	2.27
	Zewde [38]	Insuffiency of medical staff	
		Delay in making diagnosis	
		Poor communication during referral	
Emergency medicine	Aikpitanyi [14]	Delay in deciding to refer patients	5.6
	Haddad [20]	Difficulty in communication between hospital and regulatory centre	18.8
		Delay in referral/transfer	5.2
	Mahmood [29]	Failure in delay and emergency response	42.9
		Delay in procedures	28.6
		Lack of policy, protocol and guidelines.	46.4
		Delay in emergency response	33.3
		Lacking knowledge and skills	60
		Failure to follow best practice	70
		Lack of recognition of seriousness.	50
	Sayinzoga [35]	delay of the ambulance to reach the health centre	
	Nasorro [32]	Inadequate preparation in complete readiness	17
Management	Aikpitanyi [14]	Lack of skilled manpower	11.1
	Mahmood [29]	Inadequate access to senior clinical staff	39.3
		Failure to seek supervision or help	43.3
	Sayinzoga [35]	Delay in referring the patient at high level	
	Sorensen [36]	Staff not available	
	Nasorro [32]	Delated referral from another facility	26
	Saucedo [34]	Lack of 24/7 on-site presence of obstetrician or anesthesiologist	5/66 28/81 obstetrician
			13/66 37/81 anesthesiologist
	Zewde [38]	Unavailability of a senior obstetrician	
		Inappropriate management	
		Multiple referrals between health facilities	
Health workforce	Johansen [24]	Failure in teamwork	14
	Johansen [25]	Failure in cooperation between midwife and physician	16
Pharmaceuticals and medical products	Aibar [13]	Peripheral venous catheter	86.2
		Closed bladder catheter	18.9
	Aikpitanyi [14]	Non-availability of blood products	33.3
		Lack of essential emergency drugs	11.1
	Benimana [15]	Delayed or lacking supplies (blood and medication)	5.8
	Haddad [20]	Lack of medication	1.8
		Absence of blood products	1.3
	Johansen [24]	Failure in administration of medication	11.1
	Sayinzoga [35]	Lack of isogroup blood	
	Wasim [37]	Inadequacy in blood arrangement	
	Zewde [38]	Lack of supplies and equipment	
Health Facilities	Aikpitanyi [14]	Non-functional ICU	11.1
	Carvalho [16]	Inadequate prenatal care: difficult access due to lack of specialised services	46.5
	Mulongo [31]	Lack of continuity of care and coordination	
	Wasim [37]	Inadequacy in overburdened ICU	
Information Systems	Iwuh [22]	Incomplete registration (lack of information)	6.3
	Johanssen [24]	Failure in documentation	5
	Huner [21]	Lack of documentation	

**Table 4:** Contributing factors categorised and summarised.

	Protocols		Delay			Equipment and Staff		Communication	
	Presence of Adequate Protocol	Not Following Protocol	Delay in Referral/Transfer	Delay in Diagnostics	Delay in Decision-Making/Therapy	Lack of Equipment	Lack of (Well Trained) Staff	Verbal	Medical File
Individual healthcare worker		5.9% [18] 70% [24]	0.9% [21] 5.2% [20] 5.6% [14] 26% [32]	4.5% [22] 13.7% [20] 17% [25] 36.4% [21] 41.3% [15]	5.8% [15] 6.5% [20] 27.8% [14] 28.6% [20] 33.3% [29] 44.3% [21] 46.0% [24] 48% [25] 61% [32]		13% [32] 18.2% [21] 56% [25] 60% [29]	1.6% [18] 6.25% [22]	5% [24] 6.3% [22]
Teamwork							14% [24] 39% [25] 43.3% [29]	10% [18]	
System	46.4% [29]			42.9% [29]		6.9% [18] 55.5% [14] 5.8% [15] 3.1% [20]	2.6% [18] 5.1% [20] 11.1% [14] 28% [34] 39.3% [29]	18.8% [20]	14.5% [18]

## Data Availability

All data supporting the findings of this study are available within the article and its supplementary materials.

## Supplementary Materials

The following supporting information can be downloaded at: https://www.mdpi.com/article/10.3390/healthcare11111636/s1, Table S1. Summary of findings (PDF).

## Appendix A

**Table A1:** The specific search terms including used filters, and the number of articles found per database).

Search Database	Search Terms	Filters	Articles (*n*)
Pubmed	Medical error, medical mistake, adverse event, risk management, health care quality assessment, health care quality, access, and evaluation, gynaecology and/or obstetrics	Language: English or Dutch Full text Last 10 years * Title/abstract gynaecology and/or obstetrics	1598
EMBASE	Medical error, medical mistake, adverse event, complication, risk factor, risk management, incident report, risk report, quality assurance, quality assessment, health care quality assessment, health care quality, access, and evaluation, gynaecology or obstetrics	Language: English or Dutch Last 10 years	701
Web of knowledge	Medical error, medical mistake, adverse event, complication, risk factor, risk management, incident report, quality assurance, quality assessment, health care quality assessment	Language: English or Dutch Timespan: 2010–2020	2988
cocchrane library	Medical error, medical mistake, adverse event, risk management, health care quality assessment, health care quality, access and evaluation, gynaecology, obstetrics	Jan 2010–Jan 2020	1003
Total (before exclusion)			6290

* Inter observer reliability.
